# Relationship of ABO blood groups with metabolic syndrome and its metabolic abnormalities in a population in North Lahore

**DOI:** 10.12669/pjms.42.9.13447

**Published:** 2026-09

**Authors:** Mohammad Perwaiz Iqbal, Hijab Sarfraz Ahmad, Saleem Perwaiz Iqbal, Muhammad Khurram

**Affiliations:** 1 Mohammad Perwaiz Iqbal Department of Life Sciences, Department of Biological and Biomedical Sciences, Aga Khan University, Karachi, Pakistan, University of Management and Technology, Lahore, Pakistan; 2 Hijab Sarfraz Ahmad Department of Life Sciences, University of Management and Technology, Lahore, Pakistan; 3 Saleem Perwaiz Iqbal Department of Community Medicine, Shalamar Medical and Dental College, Lahore, Pakistan; 4 Muhammad Khurram Department of Life Sciences, University of Management and Technology, Lahore, Pakistan

**Keywords:** ABO blood groups, Hypertension, Hypertriglyceridemia, Hyperglycemia, Low HDL Cholesterol, Metabolic Syndrome, Obesity, Urban Population

## Abstract

**Objectives:**

To examine the relationship of ABO blood groups with metabolic syndrome (MetS) and its metabolic abnormalities in an urban population in Lahore.

**Methodology:**

A cross-sectional study was carried out at the Department of Life Sciences, University of Management and Technology, Lahore from August 2022 to August 2024 on 890 healthy subjects recruited from localities in Northern Lahore with informed consent. Blood samples were analyzed for ABO blood groups and three metabolic abnormalities, namely: fasting hyperglycemia, hypertriglyceridemia and low HDL cholesterol. In addition, central obesity and blood pressure were determined. A brief questionnaire covering demographic characteristics was also administered. Chi-square test and logistic regression were used for determining the association between ABO blood groups and MetS and its metabolic abnormalities.

**Results:**

Adults from 18 years to 75 years had a Mean ± SD age of 40.87±11.84 years. Percentages of A, B, AB and O blood groups were 22%, 41%, 11% and 26%, respectively. No association was found between ABO blood groups and MetS (p-value=0.88), however the adjusted odds of having hypertriglyceridemia in blood group A subjects was 1.8 times higher compared to subjects with blood group AB [AOR: 1.80; 95% CI: 1.02-3.18].

**Conclusion:**

There was a lack of association between ABO blood groups and MetS. Blood group A individuals appear more vulnerable to having hypertriglyceridemia in this population.

## INTRODUCTION

Metabolic syndrome (MetS) is a chronic metabolic condition/disorder that has affected nearly a quarter of the adult population in the whole world.^1^ Those suffering from this condition have increased risk of developing cardiovascular diseases and Type-2 diabetes mellitus.^2^ The five components of MetS include: hyperglycemia, hypertension, hypertriglyceridemia, low HDL cholesterol and abdominal obesity.^2^ Since ABO blood groups are genetically predetermined, it is likely that they could be associated with MetS or some of its components which also have a genetic inheritance. Although there have been a few studies carried out in South Asia and the Middle East on the association of ABO blood groups with MetS, there has been hardly any study carried out in Pakistan to explore this relationship in the Pakistani population.^3^-^6^

All the published reports have shown conflicting results with associations of ABO blood groups with some of the metabolic components but not with MetS.^3^,^4^ Therefore, the objective of the study was to explore the relationship of ABO blood groups with MetS and its metabolic abnormalities in an urban population in the North of Lahore.

## METHODOLOGY

It was a cross-sectional study, which involved recruitment of 890 healthy adults, who were recruited from the urban Northern areas of Lahore from August 2022 to August 2024, with informed consent. Their fasting blood samples were obtained for determining the levels of serum glucose, triglycerides, and HDL cholesterol.

### Ethical Approval:

The study was approved by the Biosafety and Bioethics Committee, University of Management and Technology, Lahore (Ref: sbbc-2023-09, dated August 7, 2023).

### Inclusion criteria:

Adults in age range 18-75 years with no chronic ailment (except medically managed diabetes and hypertension) such as cancer, liver disease, kidney disease and malabsorption syndrome.

### Exclusion criteria:

It included pregnant females and those suffering from any of the above-mentioned chronic diseases.

ABO blood groups were determined by using a commercially available kit based on direct hemagglutination using anti-A, anti-B, and anti-D monoclonal antibodies (Diagast, France). A brief questionnaire comprising demographic information such as age, gender, marital status, education, and average monthly household income was also administered. Waist circumference was determined using a measuring tape, while blood pressure was monitored using a sphygmomanometer. MetS was defined according to the Harmonized Criteria as described by Alberti et al.^7^ According to these criteria, the cut-off values of the five components of MetS (i.e. metabolic abnormalities) are as follows: Waist circumference > 90 cm in males or > 80 cm in females, Triglycerides ≥ 150 mg/dL (or its equivalent), HDL-C < 40 mg/dL (or its equivalent) in males or < 50 mg/dL (or its equivalent) in females, Blood pressure ≥ 130/85 mmHg, Fasting serum glucose ≥ 125 mg/dL (or its equivalent). MetS was defined as having at least three of the five above-mentioned components. For waist circumference, WHO criteria for abdominal obesity were used.^8^ Biochemical markers such as serum glucose, triglycerides and HDL cholesterol were quantitated using commercially available kits as per manufacturers’ instructions as described in a previous publication.^9^

### Statistical analysis:

Statistical analysis was done using Statistical Package for Social Sciences (SPSS) version 26.0. Mean ± SD values were compared using independent sample t-test. Chi-square test was applied for comparing percentages of various parameters, while logistic regression was used to find out the association of blood groups with MetS and its metabolic components. A p-value of less than 0.05 was considered significant.

## RESULTS

Mean ± SD age of the recruited subjects was 40.87 ± 11.84 years. There were more females compared to males (58% vs. 42%). A greater proportion of subjects were married (Table-I). Nearly 77% of them belonged to the lower socioeconomic strata with average household income below PKR 50,000 per month. The percentages of blood groups A, B, AB and O were 22%, 41%, 11% and 26%, respectively. The proportions of MetS and its five components have been listed in Table-II. The percentage of MetS in this population was 73%, while the percentage value of abdominal obesity was 79%. Hypertriglyceridemia and low HDL cholesterol were highly prevalent. Hyperglycemia appeared to be less prevalent (47%).

**Table-I:** Sociodemographic characteristics of the sampled population (n=890).

Characteristic		n	%
Gender	Male	378	42
	Female	512	58
Marital Status	Single	207	23
	Married	579	65
	Widowed	104	12
Education	None	98	11
	Primary	87	10
	Middle	143	16
	Secondary	171	19
	Intermediate	215	24
	Graduate	159	18
	Professional degree	17	2
Average income	< PKR 30,000	378	42
	PKR 30,001-50,000	315	35
	PKR 50,001-80,000	163	18
	> PKR 80,000	34	2
Blood Type	A	191	22
	B	368	41
	AB	96	11
	O	235	26

**Table-II:** Proportion of metabolic syndrome and its components among sampled population (n=890).

		n	%
Abdominal Obesity	Normal	183	21
	Obese	707	79
Triglycerides	Normal	224	25
	High	666	75
HDL-C	Normal	270	30
	Low	620	70
Fasting blood glucose	Normal	474	53
	Raised	416	47
Blood pressure	Normal	380	43
	High	510	57
MetS	Absent	238	27
	Present	652	73

The association of demographic factors and blood types with MetS and its five components is shown in Table-III. Female subjects were significantly associated with MetS (p-value < 0.01), abdominal obesity (p-value <0.01), hypertension (p-value = 0.04) and low HDL cholesterol (p-value < 0.01). Similarly, married subjects were significantly associated with MetS, abdominal obesity, hypertension and hyperglycemia (p-values < 0.01). Subjects with education of intermediate level and above appeared to be more vulnerable to having MetS and hypertension. Individuals belonging to the very low-income groups (< 50,000 PKR) were more likely to be having MetS compared to individuals belonging to the higher income groups (p-value < 0.01). Blood groups A, B, O and AB were not found to be significantly associated with MetS (p-value = 0.88).

**Table-III:** Association of demographic factors and blood types with MetS and its components (n=890).

		Total	MetS (n=652)	Abd Obesity (n=707)	High BP (n=510)	High FBS (n=416)	High TG (n=666)	Low HDL-C (n=620)
Gender	Male	378	252 (39)	265 (38)	232 (46)	179 (43)	293 (44)	188 (30)
	Female	512	400 (61)	442 (62)	278 (54)	237 (57)	373 (56)	432 (70)
	p-value		< 0.01	< 0.01	0.04	0.75	0.11	< 0.01
Marital Status	Single	207	117 (18)	148 (21)	72 (14)	50 (12)	154 (23)	134 (22)
	Married	579	438 (67)	462 (65)	343 (67)	286 (69)	428 (64)	418 (67)
	Widowed	104	97 (15)	97 (14)	95 (11)	80 (19)	84 (13)	68 (11)
	p-value		< 0.01	< 0.01	< 0.01	< 0.01	0.33	0.08
Education	None	98	74 (11)	79 (11)	54 (11)	52 (12)	73 (11)	80 (13)
	Primary	87	55 (8)	70 (10)	41 (8)	39 (9)	58 (9)	65 (10)
	Middle	143	103 (16)	118 (17)	75 (15)	56 (14)	101 (15)	116 (19)
	Secondary	171	111 (17)	124 (18)	79 (16)	78 (19)	121 (18)	115 (19)
	Intermediate	215	166 (26)	173 (24)	133 (26)	97 (23)	171 (26)	145 (23)
	Graduate	159	131 (20)	129 (18)	118 (23)	85 (20)	130 (20)	86 (14)
	Profess degree	17	12 (2)	14 (2)	10 (2)	11 (3)	12 (2)	13 (2)
	p-value		< 0.01	0.37	< 0.01	0.92	0.04	< 0.01
Ave income	< PKR 30,000	378	253 (39)	293 (41)	181 (36)	158 (38)	263 (40)	279 (45)
	PKR 30,001-50,000	315	239 (37)	251 (36)	198 (39)	149 (36)	244 (37)	200 (32)
	PKR 50,001-80,000	163	135 (21)	138 (20)	112 (22)	91 (22)	134 (20)	116 (19)
	> PKR 80,000	34	25 (4)	25 (4)	19 (4)	18 (4)	24 (4)	25 (4)
	p-value		< 0.01	0.23	< 0.01	0.02	0.01	0.03
Blood Type	A	191	141 (22)	156 (22)	103 (20)	93 (22)	150 (22)	126 (20)
	B	368	271 (42)	289 (41)	218 (43)	174 (42)	280 (42)	260 (42)
	AB	96	67 (10)	71 (10)	57 (11)	43 (10)	65 (10)	66 (11)
	O	235	173 (26)	191 (27)	132 (26)	106 (26)	171 (26)	168 (27)
	p-value		0.88	0.39	0.63	0.86	0.19	0.61

Table-IV depicts the adjusted odds ratios and the 95% confidence intervals (AORs, 95% CI) for association of ABO blood groups with MetS and its five components with respect to demographic variables. With one year increase in age, the adjusted odds of MetS, abdominal obesity, high blood pressure, high fasting serum glucose levels, high concentrations of serum triglycerides increase by 1.11, 1.07, 1.11, 1.08 and 1.03, respectively. The adjusted odds of MetS abdominal obesity and low HDL cholesterol among females were 2.88, 3.72 and 5.78 times higher compared to males. Similarly, the adjusted odds among widowed subjects were 4.42 times higher for hypertension and 2.81 times higher for hyperglycemia compared to single subjects. The adjusted odds among individuals with education of intermediate level and above were 2.44-3.04 times higher for having MetS compared to no education. Regarding blood groups, the adjusted odds of having hypertriglyceridemia were 1.8 times higher in blood Group A subjects compared to blood type AB subjects [AOR: 1.80, 95% CI (1.02-3.18)].

**Table-IV:** Adjusted ORs (95% CI) depicting the association of ABO blood groups with MetS and its components.

		Adjusted ORs (95% CI)*					
		MetS (n=652)	Abd Obesity (n=707)	High BP (n=510)	High FBS (n=416)	High TG (n=666)	Low HDL-C (n=620)
Age		1.11 (1.09 - 1.14)	1.07 (1.05 - 1.09)	1.11 (1.09 - 1.13)	1.08 (1.06 - 1.10)	1.03 (1.02 - 1.05)	0.99 (0.98 - 1.01)
Gender	Male	1.00	1.00	1.00	1.00	1.00	1.00
	Female	2.88 (1.99 - 4.15)	3.72 (2.55 - 5.42)	0.88 (0.63 - 1.23)	1.11 (0.81 - 1.51)	0.86 (0.62 - 1.20)	5.78 (4.15 - 8.06)
Marital Status	Single	1.00	1.00	1.00	1.00	1.00	1.00
	Married	1.09 (0.72 - 1.65)	0.83 (0.54 - 1.29)	1.33 (0.89 - 1.99)	1.53 (1.02 - 2.29)	0.76 (0.51 - 1.15)	1.30 (0.85 - 1.98)
	Widowed	1.98 (0.79 - 5.01)	1.58 (0.62 - 4.03)	4.42 (1.92 - 10.13)	2.81 (1.49 - 5.32)	0.77 (0.39 - 1.37)	1.08 (0.57 - 2.07)
Education	None	1.00	1.00	1.00	1.00	1.00	1.00
	Primary	0.79 (0.37 - 1.68)	1.48 (0.66 - 3.31)	1.02 (0.50 - 2.08)	0.96 (0.49 - 1.87)	0.70 (0.36 - 1.37)	0.79 (0.36 - 1.71)
	Middle	1.43 (0.71 - 2.90)	1.85 (0.89 - 3.86)	1.39 (0.73 - 2.65)	0.79 (0.43 - 1.44)	0.89 (0.48 - 1.62)	1.24 (0.60 - 2.57)
	Secondary	1.36 (0.69 - 2.67)	1.17 (0.59 - 2.31)	1.59 (0.84 - 2.99)	1.55 (0.86 - 2.80)	0.97 (0.53 - 1.77)	0.51 (0.26 - 1.01)
	Intermediate	2.44 (1.21 - 4.90)	1.80 (0.90 - 3.63)	2.79 (1.48 - 5.26)	1.26 (0.70 - 2.26)	1.35 (0.73 - 2.48)	0.58 (0.29 - 1.14)
	Graduate	3.04 (1.43 - 6.50)	1.64 (0.78 - 3.44)	6.05 (3.01 - 12.17)	1.73 (0.93 - 3.21)	1.48 (0.76 - 2.86)	0.22 (0.11 - 0.45)
	Profess degree	1.03 (0.27 - 3.92)	1.55 (0.35 - 6.89)	2.48 (0.70 - 8.81)	2.82 (0.83 - 9.65)	0.81 (0.24 - 2.79)	0.39 (0.10 - 1.54)
Ave income	< PKR 30,000	1.00	1.00	1.00	1.00	1.00	1.00
	PKR 30,001-50,000	1.25 (0.84 - 1.87)	1.08 (0.71 - 1.64)	1.24 (0.85 - 1.80)	0.99 (0.70 - 1.42)	1.24 (0.86 - 1.79)	0.87 (0.60 - 1.26)
	PKR 50,001-80,000	1.30 (0.74 - 2.26)	1.20 (0.68 - 2.12)	1.01 (0.62 - 1.63)	1.04 (0.67 - 1.64)	1.52 (0.92 - 2.51)	1.66 (1.01 - 2.74)
	> PKR 80,000	0.73 (0.28 - 1.90)	0.51 (0.20 - 1.29)	0.58 (0.24 - 1.40)	0.82 (0.35 - 1.90)	1.02 (0.43 - 2.42)	1.61 (0.65 - 4.01)
Blood Type	AB	1.00	1.00	1.00	1.00	1.00	1.00
	A	1.46 (0.78 - 2.74)	1.75 (0.93 - 3.31)	0.86 (0.47 - 1.56)	1.37 (0.79 - 2.04)	1.80 (1.02 - 3.18)	0.85 (0.47 - 1.53)
	B	1.27 (0.72 - 2.26)	1.20 (0.68 - 2.12)	1.12 (0.65 - 1.95)	1.22 (0.74 - 2.40)	1.55 (0.93 - 2.58)	0.94 (0.54 - 1.62)
	O	1.37 (0.75 - 2.53)	1.55 (0.84 - 2.87)	1.05 (0.59 - 1.87)	1.11 (0.65 - 1.91)	1.34 (0.79 - 2.29)	0.95 (0.53 - 1.70)

* Adjusted for co-variates, BP: Blood pressure, FBS: Fasting blood sugar, TG: Triglycerides, Low HDL-C: Low HDL cholesterol.

## DISCUSSION

We found no association of ABO blood groups with MetS. These results are in line with those reported in four studies carried out on South Asian and Middle Eastern populations.^3^-^6^ This could be due to the fact that in most of these studies sample size was small in those former studies. However, some of these studies have reported an association of ABO blood groups with some of the metabolic abnormalities associated with MetS. For example, Mashahit et al. found no association of ABO blood groups with MetS but did find an association of blood groups O and AB with BMI in an Egyptian population.^4^ Similarly, Shaikh et al. reported an association of ABO blood groups with lipid profile in young adults of Hyderabad, Sindh, documenting increased serum levels of triglycerides and cholesterol in B and O blood groups.^10^ In the present study, we found a lack of association of ABO blood groups with MetS but found an association of blood group A with hypertriglyceridemia in the population in Lahore with 1.8 times adjusted odds of having hypertriglyceridemia compared to subjects with blood group AB.

The overall prevalence of hypertriglyceridemia in this study was found to be 75% (Table-II) and was the highest metabolic abnormality after abdominal obesity. This is further supported by a very comprehensive study comprising 522,719 subjects from healthcare centers in four provinces of Pakistan showing severe hypertriglyceridemia in this country, which is an established risk factor for cardiovascular disease in the Pakistani population.^11^ A study carried out in Khyber Pakhtunkhwa Pakistan by Hussain et al. found hypertriglyceridemia’s prevalence to be 72.09% and a major risk factor for coronary artery disease in this population.^12^ Ijaz et al have also shown similar association of hypertriglyceridemia with cardiovascular disease in a younger Pakistani population.^13^

All these reports indicate that hypertriglyceridemia is one of the most common metabolic abnormalities in Pakistan and a risk factor for both cardiovascular disease and MetS. Chen et al in a systematic review and meta-analysis have shown an association of blood Group-A with coronary artery disease [OR: 1.14,95% CI (1.03-1.26)].^14^ This alludes to the fact that blood Group-A subjects are more likely to have metabolic abnormalities linked to the development of cardiovascular disease. This is supported by a study from Wazirali et al. who have reported an association of blood group A with increased risk of coronary heart disease in the Pakistani Population.^15^

In a population-based study, triglycerides were reported to have a causal role in coronary artery disease.^16^ Similarly, Tune et al. have reported that cardiovascular events are consequences of MetS.^17^ Our study shows an association of blood Group-A with hypertriglyceridemia which is very common in the Pakistani population, therefore it can be conjectured that blood Group-A subjects are more vulnerable to having this metabolic abnormality. Thus, blood Group-A subjects in this population are candidates for change in dietary habits to reduce the risk of hypertriglyceridemia. These dietary modifications include avoidance of oily and fried foods, sugary drinks, red meat and butter, and requirement of the use of Omega-3 fatty acids.^18^ Those having very high levels of triglycerides will be candidates for drug therapy to reduce serum triglycerides.^19^,^20^

### Strength

A high sample size is a strength of this study.

### Limitations

The recruitment was done by a convenient sample design, therefore some element of bias cannot be ruled out. Secondly, the recruitment was from localities in Northern Lahore, and the results cannot be representative of the whole of Lahore. A multicentric study, representing people from all the four provinces of Pakistan, will be needed to obtain conclusive evidence regarding the relationship of ABO blood groups with metabolic abnormalities linked to MetS and cardiovascular disease.

## CONCLUSION

There is a lack of association of ABO blood groups with MetS in a population in Lahore, however blood group A subjects had higher odds of having hypertriglyceridemia as compared to subjects with blood group AB. Dietary modifications are recommended to reduce hypertriglyceridemia in this population, especially in blood group A subjects, to decrease their risk of developing cardiovascular disease.

### Authors’ Contributions:

**MPI** and **MK:** Conceived the project.

**HSA, MK** and **MPI:** Were involved in data collection.

**MPI, HSA** and **SPI:** Were involved in data entry, statistical analysis and interpretation of the results.

**MPI, SPI** and **HSA:** Drafted the manuscript.

**MPI, MK** and **SPI:** Reviewed the manuscript.

**MPI** and **HSA:** Were responsible for accuracy and integrity of the research work.
